# Supplementary material for: Disrupted Decision-Making: EcoHIV Inoculation in Cocaine Dependent Rats
Source: Int J Mol Sci. 2022 Aug 13;23(16):9100. doi: 10.3390/ijms23169100 (PMC9409394; doi:10.3390/ijms23169100)
Supplement: Supplementary file 1 [file ijms-23-09100-s001.zip › ijms-1832588-supplementary.pdf]

Disrupted Decision-Making: EcoHIV Inoculation in Cocaine Dependent Rats

Kristen A. McLaurin, Hailong Li, Charles F. Mactutus, Steven B. Harrod and Rosemarie M. Booze *

Cognitive and Neural Science Program, Department of Psychology, University of South Carolina, Columbia, SC 29208, USA

* Correspondence: booze@mailbox.sc.edu

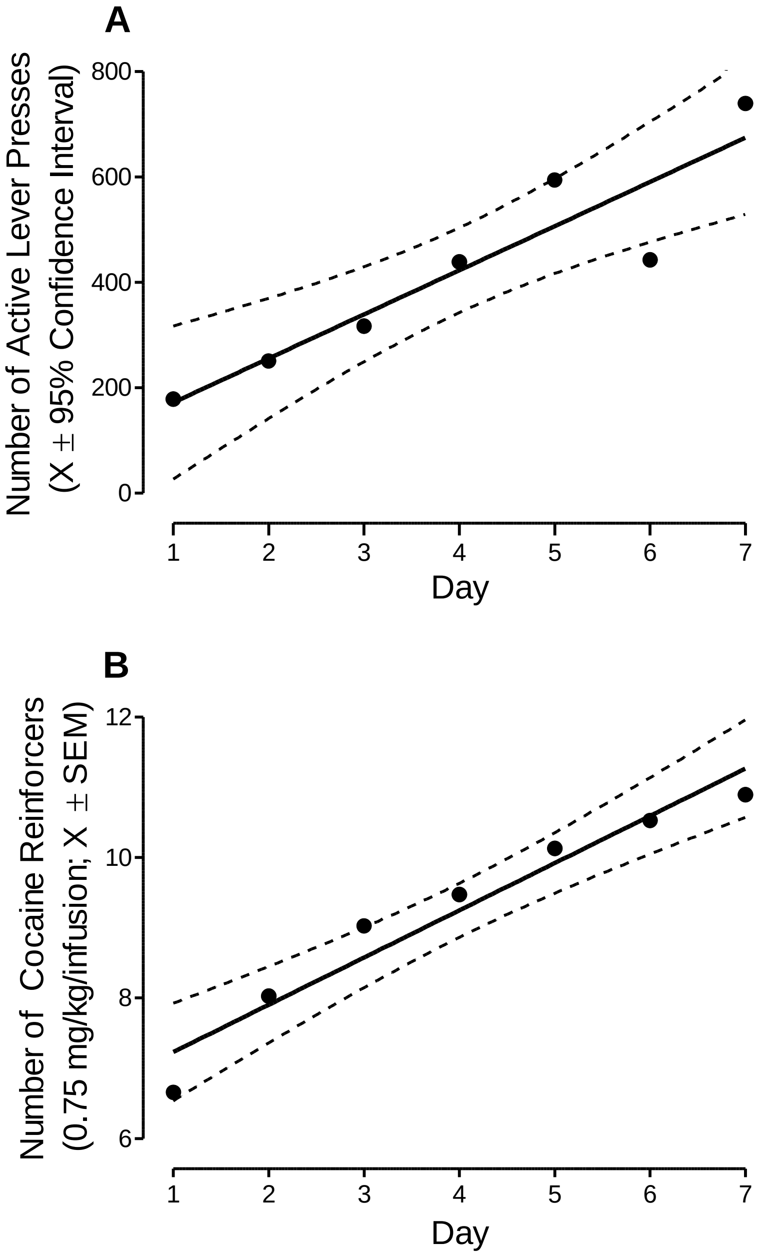

Figure S1: Cocaine Escalation. Across seven progressive ratio test sessions, animals exhibited a linear increase in the number of active lever presses (**A**) and the number of cocaine reinforcers (**B**) supporting the development of a drug dependent phenotype.

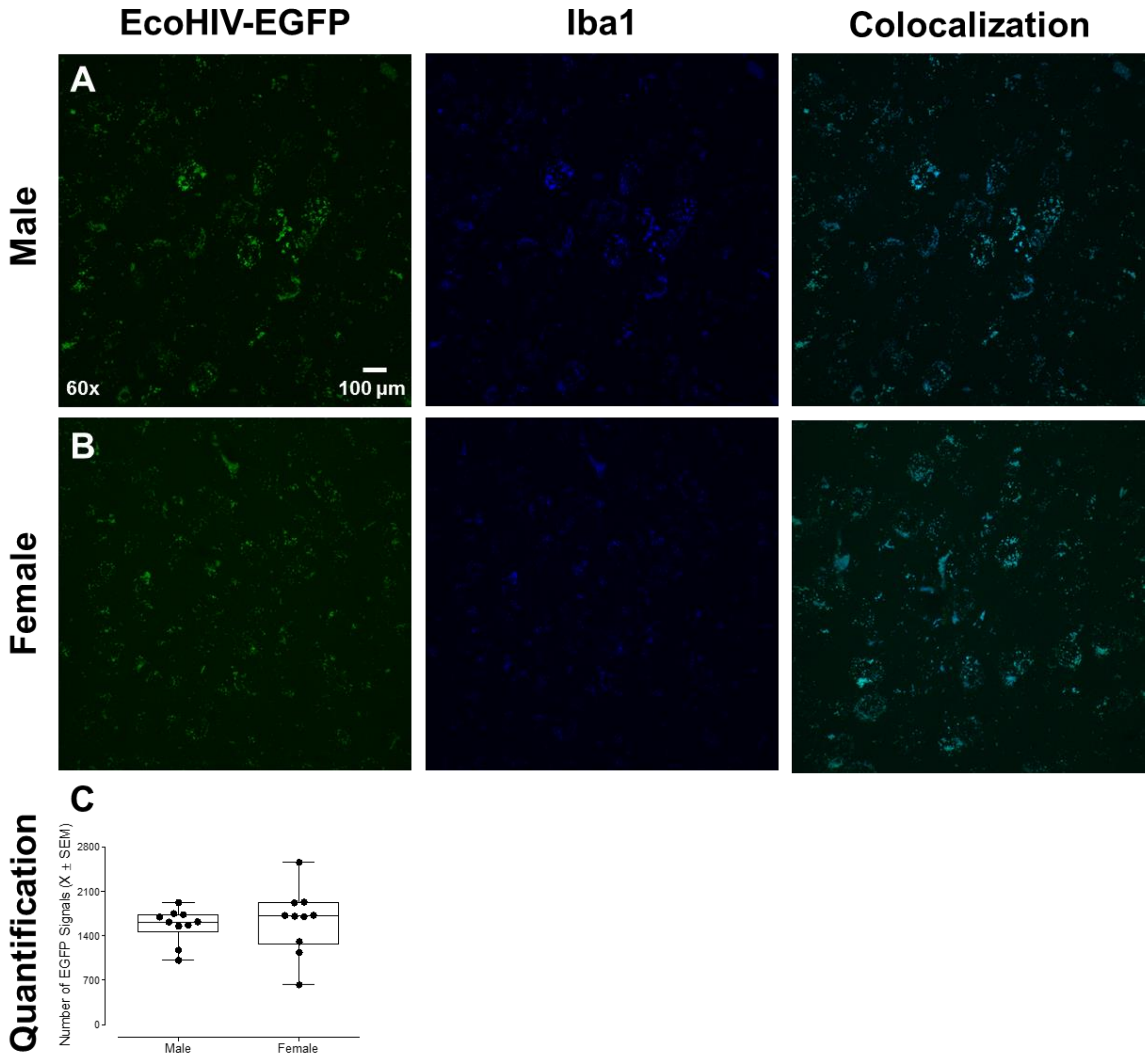

Figure S2: EcoHIV-EGFP Expression and Colocalization. Representative images of EcoHIV-EGFP, Iba1, a marker of microglia, and their co-localization in the medial prefrontal cortex (mPFC) in male (**A**) and female (**B**) animals. The number of EcoHIV-EGFP signals were quantified; no statistically significant sex differences in the number of EcoHIV-EGFP signals were observed (**C**).

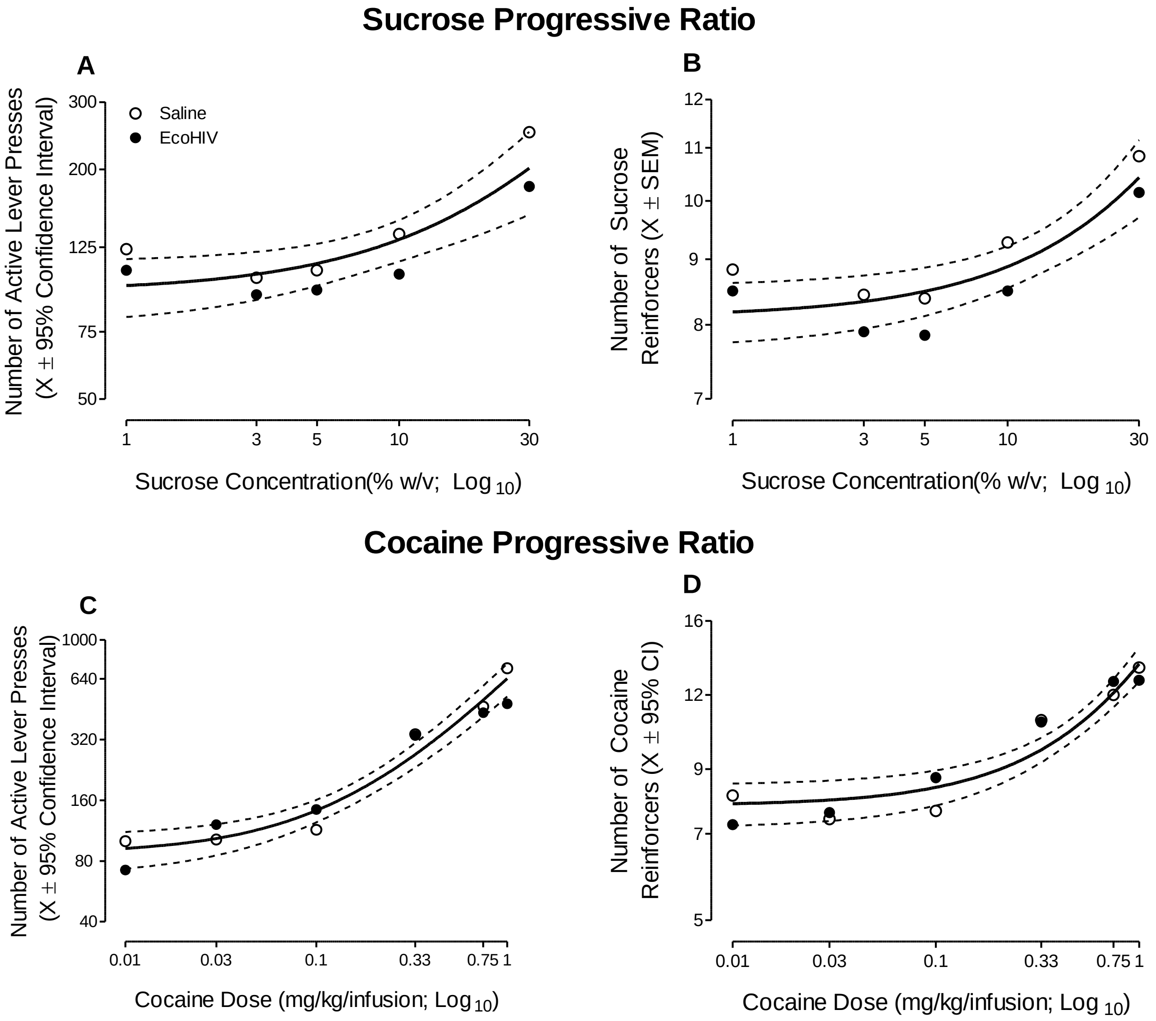

Figure S3: Reinforcing Efficacy of Sucrose and Cocaine. A progressive ratio dose-response experimental paradigm was used to evaluate the reinforcing efficacy of sucrose and cocaine in EcoHIV and saline animals. EcoHIV inoculation failed to alter the reinforcing efficacy of either sucrose (**A**,**B**) or cocaine (**C**,**D**) evidenced by global best-fit functions for both the number of active lever presses and number of reinforcers.

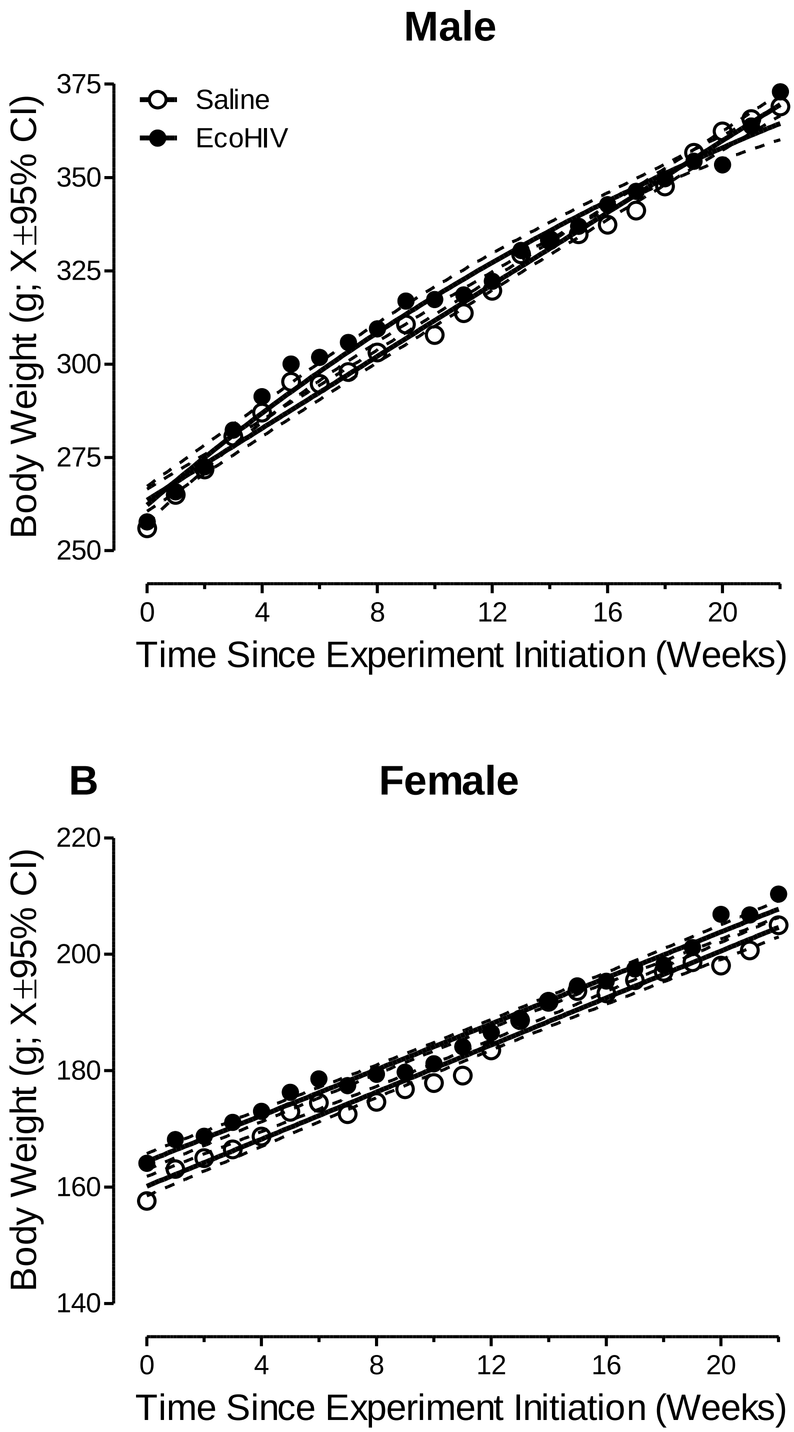

Figure S4: Body Weight. Mean body weight, a measure of somatic growth, is illustrated for male (**A**) and female (**B**) animals as a function of genotype (EcoHIV vs. Saline). All animals, independent of genotype, exhibited significant growth throughout the duration of the study. EcoHIV inoculation, therefore, had no adverse effects on somatic growth.
